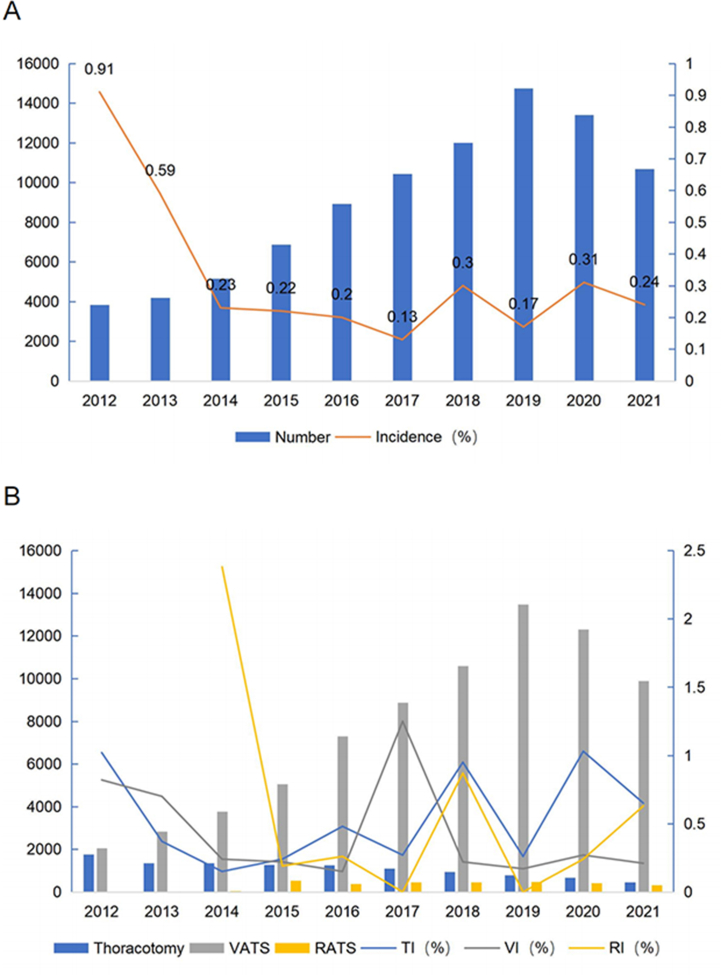# Unplanned reoperation after pulmonary surgery: Rate, risk factors and early outcomes at a single center

**DOI:** 10.1016/j.heliyon.2023.e20538

**Published:** 2023-09-30

**Authors:** Kuan Xu, Ermei Xie, Yilv Lv, Wei Gu, Minjun Shi, Jueya Yao, Jingxiang Wu, Bo Ye

**Affiliations:** a Department of Thoracic Surgery, Shanghai Chest Hospital, Shanghai Jiao Tong University School of Medicine, Shanghai, 200030, China; b Key Laboratory of Synthetic Biology Regulatory Element, Institute of Systems Medicine, Chinese Academy of Medical Sciences and Peking Union Medical College, Beijing, China; c Suzhou Institute of Systems Medicine, Suzhou, China; d Department of Anesthesiology, Shanghai Chest Hospital, Shanghai Jiao Tong University School of Medicine, Shanghai, 200030, China; e Department of General Surgery Department, Shanghai Construction Group Hospital, Shanghai, 200030, China

**Keywords:** Reoperation, Thoracic surgery, Treatment outcome

## Abstract

**Background:**

Unplanned reoperation is a potential risk factor for worse prognoses and reflects the quality of surgical treatment. This study compared the short-term outcomes between patients with and without reoperation and identified clinical factors predicting reoperation within 90 days following pulmonary surgery.

**Methods:**

Consecutive patients undergoing pulmonary resection from January 2012 to August 2021 at our institution were retrospectively reviewed. Clinical and operation-related data were collected and analyzed. Kaplan‒Meier, Cox hazard proportional regression, and propensity score matching were adopted for prognostic evaluation.

**Results:**

A total of 90263 patients were included: 247 (0.27%) patients required reoperation within 90 days. Patients undergoing unplanned reoperation had higher mortality and more postoperative complications than the nonreoperation group. Reoperation within 24 h was associated with reduced odds of mortality relative to reoperation beyond 24 h. Independent risk factors for unplanned reoperation were male sex, benign lung disease, specific surgical locations, lobectomy, and pneumonectomy. A history of smoking, pulmonary tuberculosis, intraoperative pleural adhesion, and postoperative complications were also identified as predisposing factors. The most common complication was hemorrhage in 75.7% (187 of 247).

**Conclusion:**

Our study found that unplanned reoperation was a rare but serious event that increased the risk of postoperative complications and mortality. We identified several risk factors that could be used to stratify patients according to their reoperation risk and suggest that high-risk patients should receive more intensive monitoring and preventive measures. Moreover, our study indicated that reoperating within 24 h could improve the outcomes for patients who needed reoperation.

## 1 Introduction

The widespread use of computed tomography (CT) imaging has led to the increasing detection of pulmonary diseases, including pulmonary nodules, and pulmonary resection is a primary treatment modality [1]. However, pulmonary resection carries a substantial risk of postoperative complications that may necessitate fatal outcomes and unplanned reoperations, resulting in significant financial burdens and adverse impacts on patient outcomes [2]. Unplanned reoperation refers to the repetition of the surgical procedure due to complications or untoward outcomes related to the initial surgery [3] and is considered a reliable quality indicator of a hospital's ability to detect and manage surgical complications [4]. Accordingly, improving patient outcomes by ameliorating unplanned reoperation is a significant goal in advancing lung surgery.

The incidence of unplanned reoperation after lung surgery ranges from 1.01% to 1.09% [2,5], while it varies between 0.27% and 1.76% in lung cancer surgery [[6], [7], [8]]. One study reported a decreasing trend of reoperation rate over time [8]. Though minimal access lobectomy has been associated with a lower complication rates than open access [2,9], the association between video-assisted thoracoscopic surgery (VATS) and robot-assisted thoracoscopic surgery (RATS), which have become more popular and widely implemented in lung surgery in recent decades [10], and the reduction of re-exploration is not well established. Several predictive factors of severe complications requiring reoperation, such as lung or pleural infections, recent antiplatelet drug administration, and pneumonectomy, have been identified in general thoracic surgery [11]. Patient characteristics such as older age, male sex, and preoperative radiation are risk factors for repeat oncologic thoracic surgery [8]. However, reports about the impact of potential operation-related characteristics, such as pleural adhesion, surgery timing (daytime vs. night-time), and nodal dissection strategies, on the need for reoperation are lacking. Therefore, we aimed to reassess the unplanned reoperation rate after lung surgery and to categorize and quantify the reasons for reoperation in modern lung surgery. We also explored more potential risk factors for reoperation and analyzed the outcomes of lung surgery in a matched cohort from a high-volume thoracic center.

## 2 Methods

### 2.1 Patient selection

This study was a retrospective, single-center, and cohort study conducted in accordance with the Declaration of Helsinki amendments of 2013. We evaluated patients who underwent lung resection through various surgical procedures between January 2012 and August 2021 at Shanghai Chest Hospital. Resection procedures include lobectomy, segmentectomy, wedge resection, pneumonectomy, sleeve resection and bullectomy, which may or may not involve lymph node dissection. Access procedures include VATS and RATS, which are minimally invasive techniques that use small incisions and specialized instruments. In VATS, either double or single incisions are made, and hemostasis is achieved by using ultrasonic scalpel and argon plasma coagulation. Patients who underwent biopsy, extended resection combined with chest wall excision, or lung transplantation or had incomplete data were excluded. The present study ultimately included 90,263 consecutive patients with malignant or benign diseases. Unplanned reoperation is defined as a repeat operation within 90 days due to complications or undesirable outcomes related to the initial surgery. This retrospective study was approved by the Institutional Review Board of Shanghai Chest Hospital, China (Number: IS22096).

### 2.2 Clinicopathologic evaluation

To identify risk factors for reoperation, we collected clinical characteristics, including patient age, sex, body mass index (BMI), primary diagnosis, and surgical details, such as the approach, type of resection, and location. To explore the potential comorbidities and operative mechanisms associated with complications requiring reoperation, we performed propensity score matching (PSM) to balance the above demographic characteristics between the reoperation and nonreoperation groups. Smoking history and Charlson comorbidity index (CCI) scores excluding age [12] were assessed in the matching group. The American Society of Anesthesiologists Physical Status (ASA PS) was evaluated by an anesthetist and ranged from 1 to 5 [13]. We also evaluated the anemia status, creatinine level, induction therapy, anticoagulant therapy and D-dimer levels (≥0.5 mg/l vs. <0.5 mg/l). Typically, we recommend cessation of smoking 2 weeks prior to surgery for smoking patients. And for patients receiving administration of antiplatelets or anticoagulants, we discontinue these therapies 3–5 days (vitamin K antagonists) or 5–7 days (antiplatelet therapy) prior to surgery to allow the anticoagulant effect to dissipate or to allow full normalization of platelet function.

The operative variables included surgery duration, diffuse pleural adhesion, lymph node dissection scope (none, hilar, mediastinal), intraoperative blood loss and extended scope of lung resection. Pleural adhesion that prevented the lung from fully expanding or collapsing and possibly caused by tumor was separated by ultrasound knife in our institution. The postoperative data comprised hospitalization time, the occurrence of postoperative complications, unanticipated readmission and mortality within 90 days. Only complications of grade II or higher according to the Clavien–Dindo classification [14] were recorded. The decision to perform a reoperation depended on the severity of the complications and patient's condition. For example, indications of re-exploration for bleeding included shock with tachycardia and/or hypotension, chest bloody fluid drainage (CBFD) exceeding 200 mL/h for 3 h, failure of drug-coagulation therapy. Reoperation was performed after unsuccessful drainage and antimicrobial treatment in bronchopleural fistula presenting with empyema, cutaneous emphysema, and respiratory failure.

For patients who underwent reoperation, we collected the timing of their return to the operating room, complications after reoperation, hospitalization time, unanticipated readmission and mortality within 90 days. Then, we divided patients who underwent reoperation into early reoperation and late reoperation groups based on the timing of reoperation after initial surgery (within or beyond 24 h) to compare their prognosis.

### 2.3 Statistical analysis

Demographic and clinicopathologic data are presented as medians (interquartile ranges) for continuous variables and numbers (percentages) for categorical data. We analyzed associations between variables using Student's *t*-test or the Mann‒Whitney *U* test for continuous variables and the chi-squared test for categorical variables. Short-term follow-up was calculated from the date of initial resection or reoperation to the date of death from any cause or the 90th follow-up. All *P* values were based on two-tailed statistical analysis, and *P* values less than 0.05 were considered significant. Multivariate analysis was performed using the logistic regression model to assess the predictive value of each factor found to be significant in the univariate analysis (*P* < 0.05). For PSM between the reoperation group and nonreoperation group, 1:4 PSM without replacement was implemented to balance patient age, sex, BMI, primary diagnosis, surgical approach, type of resection, location and year of surgery in RStudio (version 4.2.1, The R Foundation, Vienna, Austria) with the R packages “MatchIt” version 4.4.0 and “foreign” version 0.8–82, and the nearest neighbor matching method with a caliber of 0.01 was used. Other analyses were performed with SPSS 26.0 software (IBM Corporation, Armonk, NY, USA).

## 3 Results

### 3.1 Patient characteristics

**Fig. 1:**
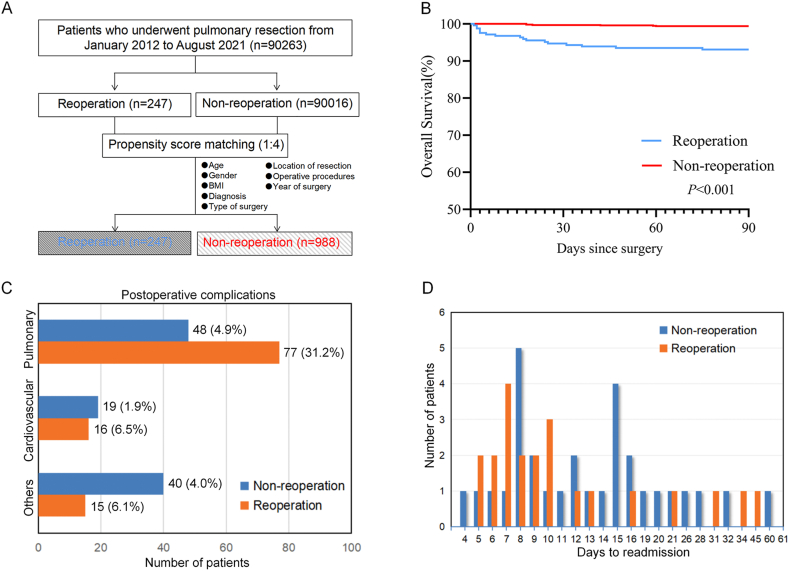
Patient selection and early outcome of the matched patients. A. Patient selection scheme. B. 90-day overall survival of patients with or without reoperation in the matched cohort. C. Postoperative complications of patients in the matched cohort. D. Time from discharge to readmission.

We included 90,263 patients who underwent pulmonary resection at our institution between January 2012 and August 2021 in our study. Of these, 247 (0.27%) required unplanned reoperations (Fig. 1A). We compared the general characteristics of the reoperation and nonreoperation groups (Table 1). The reoperation group was older (median age: 61 vs. 59 years, *P* = 0.018) and had more male patients (78.1% vs. 44.2%, *P* < 0.001) than the nonreoperation group. The reoperation group also had more thoracotomies, pneumonectomies, and right lower lobe resections. Moreover, BMI, diagnosis, and surgical year differed significantly between the two groups. To investigate the effects of comorbidities and other intraoperative and postoperative factors, we applied PSM to balance all the characteristics between the groups. After matching, there were no significant differences in any of the variables mentioned above between the reoperation and nonreoperation groups (Table 1). Supplementary Table 1 shows a comparison of comorbidities and operation-related factors between the matched groups. The reoperation group had higher rates of smoking history, pulmonary tuberculosis history, and elevated D-dimer. Furthermore, the reoperation group had more pleural adhesion, mediastinal nodal dissection, blood loss, transfusions, surgical duration, and specimen size than that of the nonreoperation group. They also had longer hospital stays and more postoperative complications.

**Table 1:** Baseline patient characteristics of patients with and without reoperation before and after matching.

Variables	Original cohort (n = 90263)			Matched cohort (n = 1235)		
	Reoperation (n = 247)	Nonreoperation (n = 90016)	*P*	Reoperation (n = 247)	Nonreoperation (n = 988)	*P*
Age(years)	61(52–67)	59(50–66)	**0.018**	61(52–67)	62(56–66)	0.123
Sex			**<0.001**			1.000
Male	193(78.1)	39808(44.2)		193(78.1)	772(78.1)	
Female	54(21.9)	50208(55.8)		54(21.9)	216(21.9)	
BMI	23.57(±2.93)	22.90(±3.04)	**0.001**	23.57(±2.93)	23.32(±2.98)	0.221
Diagnosis			**<0.001**			0.263
Malignant disease	206(83.4)	80381(89.3)		206(83.4)	832(84.2)	
Pulmonary tuberculosis	16(6.5)	484(0.5)		16(6.5)	48(4.9)	
Bronchiectasis	7(2.8)	259(0.3)		7(2.8)	21(2.1)	
Pulmonary bulla	7(2.8)	1377(1.5)		7(2.8)	16(1.6)	
Others	11(4.5)	7515(8.3)		11(4.5)	71(7.2)	
Type of surgery			**<0.001**			0.787
VATS	179(72.5)	76024(84.5)		179(72.5)	734(74.3)	
RATS	10(4)	3088(3.4)		10(4.0)	42(4.3)	
Thoracotomy	58(23.5)	10904(12.1)		58(23.5)	212(21.5)	
Location of resection			**<0.001**			0.846
LU	64(25.9)	21448(23.8)		64(25.9)	241(24.4)	
LL	32(13.0)	10638(11.8)		32(13.0)	121(12.2)	
RU	73(29.6)	27201(30.2)		73(29.6)	319(32.3)	
RM	8(3.2)	5747(6.4)		8(3.2)	46(4.7)	
RL	51(20.6)	11949(13.3)		51(20.6)	185(18.7)	
Multiple lobes	19(7.7)	13033(14.5)		19(7.7)	76(7.7)	
Operative procedures			**<0.001**			0.129
Wedge resection	23(9.3)	17619(19.6)		23(9.3)	88(8.9)	
Segmentectomy	23(9.3)	13455(14.9)		23(9.3)	96(9.7)	
Lobectomy	178(72.1)	55812(62.0)		178(72.1)	740(74.9)	
Pneumonectomy	14(5.7)	696(0.8)		14(5.7)	22(2.2)	
Sleeve resection	7(2.8)	1108(1.2)		7(2.8)	31(3.1)	
Bullectomy	2(0.8)	1326(1.5)		2(0.8)	11(1.1)	
Year of surgery			**<0.001**			0.857
2012–2013	60(24.3)	7972(8.8)		60(24.3)	211(21.4)	
2014–2015	27(10.9)	12005(13.3)		27(10.9)	107(10.8)	
2016–2017	32(13.0)	19328(21.5)		32(13.0)	145(14.7)	
2018–2019	61(24.7)	26686(29.7)		61(24.7)	243(24.6)	
2020–2021	67(27.1)	24025(26.7)		67(27.1)	282(28.5)	

BMI: body mass index; VATS: video-assisted thoracic surgery; RATS: robot-assisted thoracic surgery; LU: left upper lobe; LL: left lower lobe; RU: right upper lobe; RM: right middle lobe; RL: right lower lobe.

### 3.2 Reoperation rate

The volume of thoracic surgeries at our institution has increased over the last decade, with the exception of 2020, which was affected by the pandemic. The rate of unplanned reoperation declined from 0.91% in 2012 to 0.23% in 2014, and then stayed between 0.13% and 0.31% in the last eight years (Supplementary Fig. 1A). The number of VATS procedures increased, while the number of open thoracotomy (OT) procedures decreased. Since 2014, our hospital has performed RATS about 300–500 times per year (Supplementary Fig. 1B). The rate of unplanned reoperation was higher for OT patients (0.53%) than for VATS/RATS patients (0.24%, *P* < 0.001). Although the unplanned reoperation rate for OT patients was fluctuating, it dropped for VATS patients during this period. Moreover, it is pertinent to highlight that out of 189 VATS/RATS patients, 115 individuals received a minimally invasive reoperation.

### 3.3 Short-term outcome

The majority of the 247 patients who underwent reoperation achieved recovery after the first procedure. However, seven patients needed a second reoperation due to rebleeding or other fatal complications. Among the patients who had the first reoperation, 15 died during the index stay, one died after the second reoperation, and another died after discharge. Fig. 1B shows the Kaplan‒Meier curves stratified by reoperation in the matched cohort. The unplanned reoperation group had a significantly lower 90-day overall survival (OS) (93.1% vs. 99.4%, *P* < 0.001) and a higher incidence of reoperation-related complications, mainly pulmonary and cardiovascular complications, compared to the nonreoperation group (Fig. 1C). Hemorrhagic shock was the cause of death for 15 out of 17 patients in the reoperation group. Two patients succumbed to severe Bronchopleural Fistula (BPF) and infection that resulted in multiple-organ failure. The nonreoperation group had fewer thoracic complications but more complications involving other organ systems. The reoperation group also had a higher rate of unplanned readmissions within 90 days of reoperation (8.9% vs. 3.0%) (Fig. 1D). Supplementary Table 2 presents the characteristics and outcomes of patients who required unplanned reoperation. Multivariate analysis using Cox hazard proportional regression revealed that late reoperation was the only risk factor for increased mortality in the reoperation group (Supplementary Table 2).

### 3.4 Risk of reoperation

**Fig. 2:**
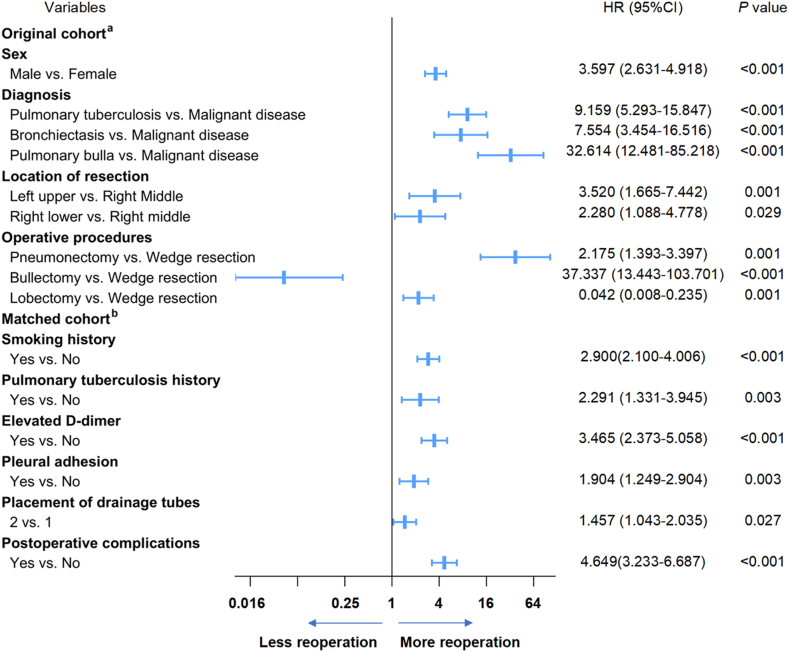
Results of multivariable analysis. a Factors associated with reoperation in multivariate analyses in the original cohort b Factors associated with reoperation in multivariate analyses in the matched cohort.

We used a multivariate logistic regression model to evaluate the preoperative risk factors for reoperation. The results indicated that male sex, pulmonary tuberculosis lesions, bronchiectasis and pulmonary bulla, right lower lobe and left upper lobe resection, lobectomy, and pneumonectomy were associated with unplanned reoperation (Fig. 2). To further assess the risk of comorbidities and other operation-related characteristics, we performed PSM to balance the groups based on age, sex, BMI, diagnosis, surgical lobe, surgical year, type of surgery, and operative procedures. In the matched cohort, multivariate analysis revealed that smoking history (HR 2.900, 95% CI: 2.100–4.006, *P* < 0.001), pulmonary tuberculosis history (HR 2.291, 95% CI: 1.331–3.945, *P* = 0.003), elevated D-dimer (HR 3.465, 95% CI: 2.373–5.058, *P* < 0.001), pleural adhesion (HR 1.904, 95% CI: 1.249–2.904, *P* = 0.003), placement of two drainage tubes (HR 1.457, 95% CI: 1.043–2.035, *P* = 0.002), and postoperative complications (HR 4.649, 95% CI: 3.233–6.687, *P* < 0.001) increased the risk of reoperation (Fig. 2). Table 2, Table 3 provide detailed information on the univariate and multivariate analyses in the original and matched cohorts.

**Table 2:** Clinical factors associated with reoperation in univariate and multivariate analyses in the original cohort.

Variables	univariate analysis			multivariate analysis		
	HR	95% CI	*P* value	HR	95% CI	*P* value
Age(years)	1.012	1.001–1.023	**0.035**	1.003	0.991–1.014	0.638
Sex			**<0.001**			**<0.001**
Female	Ref			Ref		
Male	4.508	3.333–6.097	**<0.001**	3.597	2.631–4.918	**<0.001**
BMI	1.072	1.031–1.115	**0.001**	1.033	0.990–1.078	0.139
Diagnosis			**<0.001**			**<0.001**
Malignant disease	Ref			Ref		
Pulmonary tuberculosis	13.391	7.984–22.459	**<0.001**	9.159	5.293–15.847	**<0.001**
Bronchiectasis	11.031	5.140–23.672	**<0.001**	7.554	3.454–16.516	**<0.001**
Pulmonary bulla	2.058	0.967–4.382	0.061	32.614	12.481–85.218	**<0.001**
Others	0.952	0.587–1.543	0.842	0.748	0.459–1.218	0.243
Type of surgery			**<0.001**			0.782
Thoracotomy	Ref			Ref		
VATS	0.443	0.329–0.596	**<0.001**	0.886	0.628–1.250	0.491
RATS	0.609	0.311–1.192	0.148	0.947	0.472–1.901	0.878
Location of resection			**0.001**			**<0.001**
RM	Ref			Ref		
LU	3.066	1.454–6.465	**0.003**	3.520	1.665–7.442	**0.001**
LL	2.161	0.995–4.692	0.051	2.397	1.100–5.222	0.028
RU	1.928	0.929–4.003	0.078	2.008	0.964–4.183	0.062
RL	2.144	1.027–4.473	**0.042**	2.280	1.088–4.778	**0.029**
Multiple lobes	1.047	0.458–2.394	0.913	0.428	0.151–1.209	0.109
Operative procedures			**<0.001**			**<0.001**
Wedge resection	Ref			Ref		
Segmentectomy	1.309	0.734–2.335	0.361	1.494	0.835–2.674	0.176
Lobectomy	2.443	1.582–3.773	**<0.001**	2.175	1.393–3.397	**0.001**
Pneumonectomy	15.409	7.895–30.073	**<0.001**	37.337	13.443–103.701	**<0.001**
Sleeve resection	4.840	2.072–11.303	**<0.001**	2.719	1.106–6.681	0.029
Bullectomy	1.155	0.272–4.906	0.845	0.042	0.008–0.235	**0.001**

BMI: body mass index; VATS: video-assisted thoracic surgery; RATS: robot-assisted thoracic surgery; LU: left upper lobe; LL: left lower lobe; RU: right upper lobe; RM: right middle lobe; RL: right lower lobe.

**Table 3:** Comorbidities and operation-related characteristics associated with reoperation in univariate and multivariate analyses in the matched cohort.

Variables	univariate analysis			multivariate analysis		
	HR	95% CI	*P* value	HR	95% CI	*P* value
Smoking history (yes/no)	3.234	2.424–4.316	**<0.001**	2.900	2.100–4.006	**<0.001**
Induction therapy (yes/no)	0.547	0.245–1.222	0.141			
Pulmonary tuberculosis history (yes/no)	2.811	1.747–4.520	**<0.001**	2.291	1.331–3.945	**0.003**
Anticoagulant therapy (yes/no)	0.750	0.464–1.211	0.239			
Anemia (yes/no)	0.599	0.303–1.184	0.140			
Elevated creatinine (yes/no)	0.564	0.196–1.624	0.289			
Elevated D-dimer (yes/no)	3.308	2.377–4.603	**<0.001**	3.465	2.373–5.058	**<0.001**
CCI score (≥2/<2)	0.955	0.710–1.285	0.763			
ASA PS score (≥3/<3)	1.185	0.872–1.611	0.277			
Surgery periods (night-time/working hours)	1.814	0.972–3.387	0.061			
Pleural adhesion (yes/no)	2.056	1.426–2.964	**<0.001**	1.904	1.249–2.904	**0.003**
Nodal dissection						
No dissection	Ref			Ref		
Hilar	1.178	0.692–2.005	0.547	0.908	0.494–1.667	0.755
Mediastinal, systematic or selective	2.480	1.581–3.890	**<0.001**	1.625	0.920–2.870	0.095
Length of operation (>2 h/≤2 h)	1.470	1.111–1.946	**0.007**	0.735	0.523–1.032	0.075
Blood loss (>100 mL/≤100 mL)	2.037	1.449–2.865	**<0.001**	0.988	0.652–1.497	0.953
Blood transfusion (yes/no)	0.988	0.945–1.034	0.610			
Maximal specimen diameter (>10 cm/≤10 cm)	2.241	1.576–3.186	**<0.001**	1.160	0.741–1.816	0.516
Number of drainage tubes (2/1)	2.027	1.529–2.687	**<0.001**	1.457	1.043–2.035	**0.027**
Postoperative complications (yes/no)	5.601	4.052–7.742	**<0.001**	4.649	3.233–6.687	**<0.001**

CCI: Charlson Comorbidity Index; ASA PS: American Society of Anesthesiologists Physical Status.

### 3.5 Reasons for unplanned reoperation

Hemorrhage was the leading cause (75.7%, n = 187) of unplanned reoperation, followed by bronchopleural fistula (10.1%, n = 25), chylothorax (4.5%, n = 11), and atelectasis (3.6%, n = 9). Other causes of reoperation are listed in Table 4. Most of the bleeding cases (76.5%, n = 143) had reoperation within 24 h. Among all patients, nearly half (43.3%, n = 81) presented signs of shock, such as tachycardia and hypotension, while more than a quarter (28.9%, n = 54) had CBFD exceeding 200 mL/h for 3 h. Clinical manifestations of bronchopleural fistula included empyema, cutaneous emphysema, and respiratory failure. Reoperation was performed after drainage and antimicrobial treatment failed. The bronchial artery was the main source of hemorrhage (19.3%, n = 36), especially in the early reoperation group (23.1%, n = 33). Pleural adhesion separation surfaces and intercostal vessels accounted for similar proportions of bleeding (18.2% and 17.6%, respectively). Other sites of postoperative bleeding are presented in Supplementary Table 3. We found that the bronchial artery was the most frequent site of bleeding in early reoperation patients (23.1%). However, in patients with late reoperation, the pleural adhesion separation surface was the most common site (27.3%).

**Table 4:** Causes of unplanned reoperation and postoperative characteristics after the initial operation.

Characteristic	Early Reoperation (n = 147)	Late Reoperation (n = 100)	All (n = 247)
Hemorrhage	143(97.3)	44(44.0)	187(75.7)
Shock signs and symptoms	52	29	81
Blood hemoglobin ≤90 g/L	16	12	28
CBFD >200 mL/h for 3 h	35	19	54
BPF	0(0.0)	25(25.0)	25(10.1)
Empyema		17	17
Cutaneous emphysema		5	5
Respiratory failure		5	5
Chylothorax	0(0.0)	11(11.0)	11(4.5)
Pleural effusion		11	11
Dyspnea		4	4
Atelectasis	0(0.0)	9(9.0)	9(3.6)
Positive resection margins in malignant disease	1(0.7)	2(2.0)	3(1.2)
Air leak	1(0.7)	2(2.0)	3(1.2)
Empyema	0(0.0)	3(3.0)	3(1.2)
Cardiac tamponade	2(1.4)	0(0.0)	2(0.8)
Paraplegia, clot oppress intervertebral foramen	0(0.0)	1(1.0)	1(0.4)
Pulmonary artery bronchial fistula	0(0.0)	1(1.0)	1(0.4)
Tracheoesophageal fistula	0(0.0)	1(1.0)	1(0.4)
Incarcerated incisional hernia	0(0.0)	1(1.0)	1(0.4)

CBFD: chest bloody fluid drainage; BPF: bronchopleural fistula.

## 4 Discussion

Reoperations are essential interventions for managing severe complications that may arise after surgical procedures. They are rare, but they impose an additional burden on patients and are associated with higher morbidity and mortality rates [5,7]. Our findings corroborate those of previous studies that reported unplanned reoperations as predictors of prolonged hospital stays, increased complications, and reduced short-term survival rates [2,5]. To address this issue, surgeons should be cognizant of risk factors that occur before, during, and after surgery. Understanding these factors will enable us to enhance early detection and implement appropriate preventive measures against reoperation.

The 90-day unplanned reoperation rate in our cohort was 0.27%, lower than previous studies [2,5]. This may be attributed to the increased implementation of VATS, which accounted for approximately 92% of pulmonary surgeries in 2020, and the growing expertise of our surgeons. VATS has demonstrated various advantages over thoracotomy, such as reduced trauma, pain, hospital stay, complications, and increased patient satisfaction due to its less invasive approach [9,10,15]. Despite these advantages, some challenges may arise during the VATS procedure, most notably bleeding, which is the most dangerous and direct correlate of reoperation. Our study found that 76.5% of VATS patients who underwent unplanned reoperation had hemorrhage. Intercostal vessels are frequently damaged by ports through the chest wall, and injuries to arterial branches are common during hilum dissection. Our findings revealed that these two are common bleeding sites. These sites may vary across institutions. Zheng et al. reported that the surgical incision and parietal pleura were the most frequent sources of bleeding [16]. Dai et al. reported that pleural adhesion separation surface and lymph node dissection surface were more prone to bleeding [17]. Moreover, many studies found unidentified bleeding sites in a large percentage of their cohort [6,7]. Therefore, we recommend careful inspection of all potential bleeding sources before closing the incision, especially the stumps of pulmonary and bronchial vessels, the pulmonary stapling line, and the lymph nodes dissection surface. The presence of pleural adhesions constitutes another potential risk factor for complications, not only bleeding, but also pneumothorax and the development of pleural empyema, ultimately increasing the likelihood of reoperation [18]. Thus, cautious dissection of the pleural cavity is essential to minimize tissue injury. Moreover, the appropriate choice of surgical equipment is crucial, as using instruments that do not match the patient's tissue can also increase bleeding risk, such as oversized staples for thin pulmonary tissue [19].

Regarding the indication to perform reoperation for bleeding control, we recommend imaging examinations such as chest X-rays, chest CT scans, and ultrasounds for hemothorax diagnosis since they are less prone to interference from confounding factors. For massive blood loss in a short period of time (i.e., bloody fluid drainage from the chest exceeding 200 mL/h for 3 h), reoperation needs to be carried out in time. However, we found only 54 patients (28.9%) met the criteria for surgical management of postoperative hemostasis in our study. Notably, late reoperation was the sole factor associated with high mortality risk, emphasizing the critical importance of early problem detection and timely intervention in bleeding patients [17]. Therefore, based on our institution's experience, we suggest that re-exploration should be performed promptly for patients with confirmed moderate blood loss that does not meet the above criteria but shows progressive decrease in hemoglobin.

We found that anatomic variations and comorbidities are also associated with reoperation. Performing lobectomy on the left upper lobe is notoriously difficult due to its proximity to heart and large vessels, which can easily sustain damage during VATS procedures and lead to the conversion to thoracotomy [20]. The anatomic challenges of the right main bronchus, characterized by its rigidity and shorter length in comparison to the left main bronchus, render it particularly susceptible to anastomotic tension during right lower lobectomy, consequently elevating the risk of BPF [21]. As noted in the literature, our study findings showed a significant association between bronchiectasis and reoperation [5]. Patients with bronchiectasis or bulla often experience recurrent infections that lead to significant pleural adhesions and increase the complexity of surgery [22]. A similar scenario is observed in patients with pulmonary TB or a history of TB, who often have dense pleural adhesions, sticky and enlarged lymph nodes, and broncholithiasis [23]. Furthermore, among 77 patients with high D-dimer levels in our reoperation group, 59 patients (76.6%) required reoperation due to bleeding, indicating a link between postoperative bleeding and elevated D-dimer levels [24]. It should be noted that multiple individual factors, such as smoking, excessive alcohol consumption, and overeating, can affect surgical outcomes between genders. Therefore, implementing drug management for pulmonary TB and encouraging the cessation of detrimental habits prior to surgery may confer substantial benefits to these patients.

BPF is a rare but life-threatening complication with the mortality rates ranging from 11% to 18% [25]. It most commonly occurs after lobectomy (76% of cases in our study), and certain factors increase the risk of BPF, such as infectious lung diseases, neoadjuvant radiotherapy, low BMI, and diabetes, which compromise the blood supply to the bronchial stump, culminating in suboptimal wound healing and delayed closure [21,26]. Remarkably, our findings indicate that a substantial majority of BPF patients (72%) exhibited at least one of these predisposing factors. Hence, it is advisable to diligently monitor these conditions before surgery and consider employing flap reinforcement of the bronchial stump in high-risk patients as a potential preventive measure. Signs of BPF can be vague, therefore bronchoscopy is essential because it helps confirm the diagnosis and assess the BPF site, allowing the physician to intervene selectively in patients with suspected BPF. The optimal management strategy for BPF is contingent upon the size and location, patient comorbidities, and surgeon preferences. Endoscopic therapies, such as metal or silicone stents, fibrin sealant injections, silver nitrate, and tissue adhesives, are effective for BPFs of small to medium size [27,28]. However, larger BPFs may require surgical interventions. In our clinical experience, patients with a dehiscence size exceeding 1 cm can safely undergo direct suture repair with vascularized tissue within 30 days of symptom onset. However, for patients who underwent reoperation more than 30 days after partial lung resection (n = 5), four required completion lobectomy or bilobectomy due to diminished tissue quality, significant pleural contamination or scarring and had a higher mortality rate (20% vs. 10%).

The present study has several limitations. First, it used a retrospective design due to the unpredictable nature of unplanned reoperation and its low incidence, which may inherently introduce an element of inevitable bias. Second, we did not distinguish the surgical experience among surgeons or assess its role in surgical outcomes. Studies have shown that surgeons who conducted more than 400 operations per year were found to have a lower incidence of intraoperative accidents than those who performed fewer than 400 [29]. Third, this study lacked specific details regarding various aspects, including treatment and outcomes, indications, surgical challenges, and long-term survival. This limitation hinders our comprehension of the broader clinical implications and possible confounding factors. Therefore, further studies with a broader participant pool from multiple centers and more detailed variables are necessary to confirm our findings.

## 5 Conclusion

In conclusion, this study demonstrated that unplanned reoperation after pulmonary resection was a rare event at our institution over the past decade. We identified several significant predictors of 90-day unplanned reoperation following pulmonary resection. Accurate identification and understanding of these factors may enable us to develop individualized treatment strategies that can help prevent unplanned reoperation. Furthermore, our findings suggest that early intervention may reduce the incidence and severity of complications and improve patient outcomes.

## Author contribution statement

Kuan Xu: Ermei Xie: Bo Ye: Conceived and designed the experiments; Performed the experiments; Analyzed and interpreted the data; Wrote the paper. Yilv Lv: Wei Gu: Minjun Shi: Performed the experiments; Analyzed and interpreted the data. Jueya Yao: Jingxiang Wu: Contributed reagents, materials, analysis tools or data.

## Funding statement

This work was supported by a grant from the Shanghai Clinical Key Specialty Construction Project-Thoracic Surgery Specialty (shslczdzk02102).

## Data availability statement

Data will be made available on request.

## Declaration of competing interest

The authors declare that they have no known competing financial interests or personal relationships that could have appeared to influence the work reported in this paper.

## Figures and Tables

**figs1:**