# Supplementary material for: Unplanned reoperation after pulmonary surgery: Rate, risk factors and early outcomes at a single center
Source: Heliyon. 2023 Sep 30;9(10):e20538. doi: 10.1016/j.heliyon.2023.e20538 (PMC10560774; doi:10.1016/j.heliyon.2023.e20538)
Supplement: Multimedia component 1 [file mmc1.docx]

Supplementary table 1 Clinical characteristics in the matched patients

| **Variables** | Reoperation  (n=247) | | Non-reoperation  (n=988) | *P* |  |
| --- | --- | --- | --- | --- | --- |
| Preoperative characteristics | | |  |  |  |
| Smoking history | |  |  | **<0.001** |  |
| No | | 95(38.5) | 661(66.9) |  |  |
| Yes | | 152(61.5) | 327(33.1) |  |  |
| Induction therapy | |  |  | 0.136 |  |
| No | | 240(97.2) | 938(94.9) |  |  |
| Yes | | 7(2.8) | 50(5.1) |  |  |
| Pulmonary tuberculosis history | |  |  | **<0.001** |  |
| No | | 216(87.4) | 940(95.1) |  |  |
| Yes | | 31(12.6) | 48(4.9) |  |  |
| Anticoagulant therapy | |  |  | 0.237 |  |
| No | | 225(91.1) | 874(88.5) |  |  |
| Yes | | 22(8.9) | 114(11.5) |  |  |
| Anemia | |  |  | 0.136 |  |
| No | | 237(96.0) | 923(93.4) |  |  |
| Yes | | 10(4.0) | 65(6.6) |  |  |
| Elevated creatinine | |  |  | 0.283 |  |
| No | | 243(98.4) | 960(97.2) |  |  |
| Yes | | 4(1.6) | 28(2.8) |  |  |
| Elevated D-dimer | |  |  | **<0.001** |  |
| No | | 170(68.8) | 869(88.0) |  |  |
| Yes | | 77(31.2) | 119(12.0) |  |  |
| CCI score | |  |  | 0.763 |  |
| <2 | | 166(67.2) | 654(66.2) |  |  |
| ≥2 | | 81(32.8) | 334(33.8) |  |  |
| ASA PS score | |  |  | 0.277 |  |
| <3 | | 173(70.0) | 726(73.5) |  |  |
| ≥3 | | 74(30.0) | 262(26.5) |  |  |
| Intraoperative characteristics | |  |  |  |  |
| Surgery periods | |  |  | 0.058 |  |
| Working hours | | 232(93.9) | 954(96.6) |  |  |
| Night-time | | 15(6.1) | 34(3.4) |  |  |
| Pleural adhesion | |  |  | **<0.001** |  |
| No | | 196(79.4) | 877(88.8) |  |  |
| Yes | | 51(20.6) | 111(11.2) |  |  |
| Nodal dissection | |  |  | **<0.001** |  |
| No dissection | | 25(10.1) | 186(18.8) |  |  |
| Hilar | | 41(16.6) | 259(26.2) |  |  |
| Mediastinal | | 181(73.3) | 543(55.0) |  |  |
| Length of operation | |  |  | **0.007** |  |
| ≤2h | | 124(50.2) | 590(59.7) |  |  |
| >2h | | 123(49.8) | 398(40.3) |  |  |
| Blood loss | |  |  | **<0.001** |  |
| ≤100ml | | 186(75.3) | 851(86.1) |  |  |
| >100ml | | 61(24.7) | 137(13.9) |  |  |
| Blood transfusion | |  |  | **0.009** |  |
| No | | 240(97.2) | 980(99.2) |  |  |
| Yes | | 7(2.8) | 8(0.8) |  |  |
| Maximal specimen diameter(cm) | |  |  | **<0.001** |  |
| ≤10 | | 44(17.8) | 323(32.7) |  |  |
| >10 | | 203(82.2) | 665(67.3) |  |  |
| Number of drainage tubes | |  |  | **<0.001** |  |
| 1 | | 110(44.5) | 612(61.9) |  |  |
| 2 | | 137(55.5) | 376(38.1) |  |  |
| Postoperative complications | |  |  | **<0.001** |  |
| No | | 147(59.5) | 881(89.2) |  |  |
| Yes | | 100(40.5) | 107(10.8) |  |  |
| Hospitalization time (days) | | 7(5-14) | 5(4-8) | **<0.001** |  |

CCI: Charlson Comorbidity Index; ASA PS: American Society of Anesthesiologists Physical Status

Supplementary table 2 Association between clinical characteristics and 90-day mortality in patients who required reoperation

| Variables | Dead | Alive | univariate analysis | | | multivariate analysis | | |
| --- | --- | --- | --- | --- | --- | --- | --- | --- |
|  |  |  | HR | 95% CI | *P value* | HR | 95% CI | *P value* |
| Age(≥60/<60) | 5/12 | 79/151 | 0.796 | 0.271-2.341 | 0.679 |  |  |  |
| Sex(Male/Female) | 16/1 | 177/53 | 0.209 | 0.027-1.611 | 0.133 |  |  |  |
| Diagnosis |  |  |  |  | 0.858 |  |  |  |
| Malignant disease | 13 | 193 | Ref |  |  |  |  |  |
| Pulmonary tuberculosis | 1 | 15 | 0.990 | 0.121-8.088 | 0.992 |  |  |  |
| Bronchiectasis | 1 | 6 | 2.474 | 0.277-22.114 | 0.418 |  |  |  |
| Pulmonary bulla | 1 | 6 | 2.474 | 0.277-22.114 | 0.418 |  |  |  |
| Others | 1 | 10 | 1.485 | 0.176-12.505 | 0.716 |  |  |  |
| Type of surgery |  |  |  |  | 0.267 |  |  |  |
| Thoracotomy | 7 | 51 | Ref |  |  |  |  |  |
| VATS | 10 | 169 | 0.431 | 0.156-1.190 | 0.104 |  |  |  |
| RATS | 0 | 10 | - |  |  |  |  |  |
| Location |  |  |  |  | 0.512 |  |  |  |
| LU | 8 | 56 | Ref |  |  |  |  |  |
| LL | 0 | 32 | - |  |  |  |  |  |
| RU | 2 | 71 | 0.229 | 0.046-1.147 | 0.073 |  |  |  |
| RM | 1 | 7 | 1.163 | 0.124-10.900 | 0.895 |  |  |  |
| RL | 6 | 45 | 1.086 | 0.341-3.458 | 0.889 |  |  |  |
| Multiple | 0 | 19 | 0.452 | 0.052-3.928 | 0.472 |  |  |  |
| Operative procedures |  |  |  |  | 0.973 |  |  |  |
| Wedge resection | 2 | 21 | Ref |  |  |  |  |  |
| Segmentectomy | 0 | 23 | - |  |  |  |  |  |
| Lobectomy | 13 | 165 | 0.827 | 0.174-3.923 | 0.811 |  |  |  |
| Pneumonectomy | 2 | 12 | 1.750 | 0.218-14.069 | 0.599 |  |  |  |
| Sleeve resection | 0 | 7 | - |  |  |  |  |  |
| Bullectomy | 0 | 2 | - |  |  |  |  |  |
| Smoking history(yes/no) | 14/3 | 138/92 | 3.077 | 0.860-11.010 | 0.084 |  |  |  |
| Induction therapy(yes/no) | 2/15 | 5/225 | 6.000 | 1.073-33.544 | **0.041** | 3.252 | 0.458-23.098 | 0.238 |
| Pulmonary tuberculosis history(yes/no) | 1/16 | 30/200 | 0.417 | 0.053-3.257 | 0.404 |  |  |  |
| Anticoagulant therapy(yes/no) | 0/17 | 22/208 | - |  | 0.998 |  |  |  |
| Anemia(yes/no) | 3/14 | 7/223 | 6.827 | 1.591-29.287 | **0.010** | 4.048 | 0.855-19.163 | 0.078 |
| Elevated D-Dimer (yes/no) | 6/11 | 71/159 | 1.222 | 0.435-3.433 | 0.704 |  |  |  |
| CCI score(≥2/<2) | 4/13 | 77/153 | 0.611 | 0.193-1.938 | 0.403 |  |  |  |
| ASA PS score(≥3/<3) | 5/12 | 69/161 | 0.972 | 0.330-2.865 | 0.959 |  |  |  |
| Surgery periods |  |  |  |  | 0.999 |  |  |  |
| Working hours | 17 | 215 | Ref |  |  |  |  |  |
| Night-time | 0 | 15 | - |  |  |  |  |  |
| Pleural adhesion(yes/no) | 4/13 | 47/183 | 1.507 | 0.467-4.867 | 0.493 |  |  |  |
| Nodal dissection |  |  |  |  | 0.178 |  |  |  |
| No dissection | 4 | 21 | Ref |  |  |  |  |  |
| Hilar | 3 | 38 | 0.414 | 0.085-2.030 | 0.277 |  |  |  |
| Mediastinal | 10 | 171 | 0.307 | 0.088-1.066 | 0.063 |  |  |  |
| Length of operation(>2h/≤2h) | 12/5 | 111/119 | 1.011 | 1.002-1.019 | **0.011** | 1.007 | 0.998-1.016 | 0.148 |
| Blood loss(>100ml/≤100ml) | 5/12 | 56/174 | 1.295 | 0.437-3.835 | 0.641 |  |  |  |
| Blood transfusion(yes/no) | 1/16 | 6/224 | 2.333 | 0.265-20.578 | 0.446 |  |  |  |
| Maximal specimen diameter(>10cm/≤10cm) | 15/2 | 188/42 | 1.676 | 0.369-7.606 | 0.504 |  |  |  |
| Number of drainage tubes(2/1) | 11/6 | 126/104 | 1.513 | 0.541-4.230 | 0.430 |  |  |  |
| Beyond 24 hours/within 24 hours | 13/4 | 87/143 | 5.342 | 1.688-16.903 | **0.004** | 3.917 | 1.183-12.970 | **0.025** |
| Reason for reoperation |  |  |  |  | 0.788 |  |  |  |
| Hemorrhage | 12 | 175 | Ref |  |  |  |  |  |
| BPF | 3 | 22 | 1.989 | 0.520-7.599 | 0.315 |  |  |  |
| Chylothorax | 0 | 11 | - |  |  |  |  |  |
| Atelectasis | 0 | 9 | - |  |  |  |  |  |
| Other | 2 | 13 | 2.244 | 0.453-11.107 | 0.322 |  |  |  |

VATS: video-assisted thoracic surgery; RATS: robot-assisted thoracic surgery; LU: left upper lobe; LL: left lower lobe; RU: right upper lobe; RM: right middle lobe; RL: right lower lobe; CCI: Charlson Comorbidity Index; ASA PS: American Society of Anesthesiologists Physical Status; BPF: bronchopleural fistula

Supplementary table 3 Origin of postoperative bleeding for reoperation

| Origin of bleeding | Early reoperation  (n=147) | Late reoperation  (n=100) | All  n=187 |
| --- | --- | --- | --- |
| Bronchial artery | 33（23.1） | 3（6.8） | 36（19.3） |
| Pleural adhesion separation surface | 22（15.4） | 12（27.3） | 34（18.2） |
| Intercostal vessel | 23（16.1） | 10（22.7） | 33（17.6） |
| Lung parenchyma | 15（10.5） | 6（13.6） | 21（11.2） |
| Pulmonary artery branch | 9（6.3） | 3（6.8） | 12（6.4） |
| Incision | 9（6.3） | 1（2.3） | 10（5.3） |
| Paraspinal vessel | 5（3.5） | 2（4.5） | 7（3.7） |
| Lymph node dissection surface | 5（3.5） | 0（0.0） | 5（2.7） |
| Multi-regional | 4（2.8） | 0（0.0） | 4（2.1） |
| Arcus aortae | 0（0.0） | 2（4.5） | 2（1.1） |
| Left common carotid artery branch | 1（0.7） | 0（0.0） | 1（0.5） |
| Azygos vein | 1（0.7） | 0（0.0） | 1（0.5） |
| Pulmonary vein | 1（0.7） | 0（0.0） | 1（0.5） |
| Internal mammary artery | 0（0.0） | 1（0.7） | 1（0.5） |
| Subclavian artery | 0（0.0） | 1（0.7） | 1（0.5） |
| Unknown origin | 15（10.5） | 3（6.8） | 18（9.6） |
